# Shaping interventions to address waterpipe smoking in Arabic-speaking communities in Sydney, Australia: a qualitative study

**DOI:** 10.1186/s12889-018-6270-3

**Published:** 2018-12-17

**Authors:** Rachael Kearns, Karen Gardner, Mariela Silveira, Lisa Woodland, Myna Hua, Milena Katz, Klara Takas, Julie McDonald, Ben Harris-Roxas

**Affiliations:** 1 Centre for Primary Health Care and Equity, University of New South Wales, Sydney, Australia; 2 Health Promotion Service, South Eastern Sydney Local Health District, Sydney, Australia; 3 Priority Populations, South Eastern Sydney Local Health District, Sydney, Australia; 4 Multicultural Health Service, South Eastern Sydney Local Health District, Sydney, Australia

**Keywords:** Waterpipe smoking, Tobacco smoking, Smoking prevention, Health promotion

## Abstract

**Background:**

Waterpipe smoking is a traditional method of tobacco smoking that is being increasingly practiced worldwide. However, the research evidence describing the practice and prevalence of waterpipe smoking in Australia is limited. Arabic-speaking communities residing in an area of metropolitan Sydney identified increasing rates of waterpipe smoking as a community health concern during a tobacco intervention project. A qualitative research project was conducted to explore community perceptions about waterpipe smoking and the health promotion interventions that would be acceptable to Arabic speaking communities.

**Methods:**

Participants from Arabic-speaking community groups and networks were recruited by trained bilingual community research assistants (BCRAs). Ten focus groups were conducted, eight by the BCRAs and two by the research team, and included a total of 88 participants. Notes were taken during the focus groups by the BCRAs and provided to the research team. The data was coded and managed using NVivo 11, and examined for themes and subthemes.

**Results:**

Eleven themes were identified from the data relating to the perceptions of waterpipe smoking (practices, cultural identity, acceptability, social connectedness, knowledge and perceptions of harm, trend and fashion, availability and access) and possible health promotion interventions (health information and social marketing, health education, policy and legislation, intervention target groups and messages). Waterpipe smoking was reported to be widely practiced and was related to a number of factors including feelings of cultural identity and belonging. The study highlighted the misconceptions of harm that exist in communities about the health effects of waterpipe smoking, as well as the significant role of the family in passing on the practice of waterpipe smoking. These factors should be considered in the development of health promotion interventions.

**Conclusions:**

Our findings suggest that until waterpipe smoking is perceived as a problem, community readiness for accepting health promotion interventions will be limited. Interventions should focus on debunking the myths that contribute toward a reduced perception of harm. A culturally sensitive approach, that considers the cultural connection to waterpipe smoking, should be taken toward the development and implementation of interventions.

**Electronic supplementary material:**

The online version of this article (10.1186/s12889-018-6270-3) contains supplementary material, which is available to authorized users.

## Background

Waterpipe smoking, also known as “shisha”, “hookah”, “narghile” or “arghile” is a method of tobacco use traditionally associated with eastern societies including the Mediterranean region, Southeast Asia and northern Africa [1, 2]. A waterpipe consists of a head or tobacco bowl, a body, water bowl, hose and a mouthpiece which the smoker uses to inhale [1]. The smoker inhales air which is heated by charcoal and moves through perforated aluminium foil across flavoured tobacco [2]. Smoke passes through holes in the bottom of the head into the body of the waterpipe. When the smoker inhales through the hose, air is drawn into the charcoal and through the tobacco producing smoke aerosol [1]. The smoke generated then bubbles through water in the bowl before being inhaled by the smoker [1, 2].

Waterpipe smoking is often perceived as being less harmful than other methods of tobacco use such as cigarette smoking [3]. The passage of the smoke through water, or “filtering” of the smoke, is thought to be the foundation for these misconceptions of harm [4]. However, there is accumulating evidence to indicate that waterpipe smoking causes risks and harms to health. A narrative review conducted by El-Zaatari et al. [5] on the health effects of waterpipe smoking described a number of health implications associated with its use. These included acute and long term effects on the cardiovascular and respiratory systems, such as increased heart rate, respiratory rate and blood pressure, coronary artery disease, increased carbon monoxide, emphysema and chronic obstructive pulmonary disease, as well as a number of cancers including lung, oesophageal and gastric cancer [5].

Research evidence shows that trends in waterpipe smoking are increasing internationally with the practice representing a growing portion of global tobacco use [1]. Studies describe a disturbing pattern of increasing popularity of waterpipe smoking among youth and young adults, not only in the Middle East, but in other countries and regions around the world. Data from epidemiological studies reported by Maziak et al. [6] show prevalence rates for waterpipe smoking that surpass those of cigarette smoking among school students in the Middle East and the United Kingdom, as well as among college and university students in the United States. A recent systematic review of studies reporting on the prevalence and trends of waterpipe smoking found that country-weighted regional mean prevalence estimates for past 30 day use among youth residing in the three regions, where data was available, was highest in Europe (10.6%), followed by the Eastern Mediterranean region (10.3%) and the Americas (6.8%). Ever use amongst youth in the Americas was found to be almost twice that of adults (18.3 compared to 9.6%), with Europe having the highest prevalence of ever use amongst youth (31.8%) [7]. Waterpipe has also been found to be a significantly more common method of tobacco use among school students in most Middle Eastern countries than cigarette smoking [8].

A number of factors have been identified to explain this rapid spread in use. These include the introduction of flavoured tobacco or “Massell”, the preferred form of tobacco for most waterpipe smokers, particularly youth; the Internet, mass and social media which have facilitated the dissemination and marketing of waterpipe products and; the absence of policies and regulations to address waterpipe smoking [6]. The rise of a café culture in the Middle East has also provided a natural setting for the very social dimension of waterpipe smoking [6]. Patterns of use for waterpipe smoking have been found to vary in relation to those of cigarette smoking with international studies showing that waterpipe smoking is most often an intermittent, non-daily practice [2].

With the exception of a few studies focusing on specific populations and communities, the research evidence describing the prevalence of waterpipe smoking in the Australian context is minimal [9–11]. The 2016 National Drug Strategy Household survey reported that 5% of Australian adults had previously used a waterpipe but not in the last 12 months, and that 2% had used a waterpipe in the past 12 months [12]. Other studies indicate that rates among Arabic speaking communities in Australia are much higher. A telephone survey conducted among Arabic-speakers residing in south-west Sydney in 2004 found that 11.4% of respondents reported smoking a waterpipe with 1% of respondents being daily waterpipe smokers [11]. A later survey conducted in 2010 with the Arabic-speaking community in Melbourne, Australia found that 45% of respondents had ever smoked a waterpipe with a significantly smaller proportion of respondents describing themselves as being daily (4%) or occasional (11.8%) smokers [10]. Studies that have focused on waterpipe smoking practices in Australia are also few. One qualitative study of smoking within an Arabic-speaking community in Western Sydney described waterpipe smoking as a “common and normal social activity for men and women” [13]. In addition, community consultations, conducted as part of a tobacco intervention project with another Arabic-speaking community residing in metropolitan Sydney, found that waterpipe smoking was a prevalent and accepted practice and that there was a lack of belief in the potential associated harms, particularly among young people [14].

Despite evidence that demonstrates the increasing prevalence of waterpipe smoking, particularly amongst youth and young adults, few international studies have focused on the effectiveness of health promotion interventions targeting waterpipe smoking practices. A 2015 Cochrane review on interventions for waterpipe smoking cessation identified only three randomised controlled trials of interventions [15]. A 2016 systematic review including 15 interventions focused on waterpipe smoking prevention and cessation found that although some behavioural interventions showed encouraging results, there was minimal evidence to support the effectiveness of most interventions addressing waterpipe smoking and “better quality waterpipe interventions” were needed [16]. An understanding of the “unique features” of waterpipe tobacco smoking is required for the development of treatment and policy interventions [4].

The increasing global trend of waterpipe smoking, alongside the lack of evidence for successful prevention and cessation interventions, suggests a need for further research to investigate possible health promotion approaches and interventions that could be successful in specific contexts and population groups. The research project described in this paper, aimed to explore the perceptions held by Arabic-speaking communities, from an area of metropolitan Sydney, about the extent of waterpipe use, the cultural factors underpinning its use, community concerns and knowledge of harms, and health promotion interventions for addressing waterpipe use that would be acceptable to Arabic-speaking communities. The research project was initiated following a tobacco intervention project during which Arabic-speaking communities identified increasing rates of waterpipe smoking as a community concern [14]. Whilst a number of international studies have sought to describe and understand the determinants of waterpipe smoking, there have been no Australian studies that we are aware of that have specifically researched the perceptions and cultural factors that influence the practice of waterpipe smoking amongst Arabic speaking communities in the Australian context. The findings of this research study contribute insight and understanding about the interrelated factors underpinning waterpipe use that could be considered in the development of health promotion interventions to address waterpipe smoking in Arabic-speaking communities.

## Methods

### Focus groups

Focus groups were selected as the data collection method most appropriate to the research questions which aimed to understand the diversity of perceptions held about waterpipe smoking by members of a specific community. Focus groups also allowed a larger number of community members to participate in the research and to add to each others’ responses, with the aim of capturing a broader range of experiences and perspectives during the data collection. Ten focus groups were conducted between August and October 2016 with Arabic-speaking community members residing in an area of metropolitan Sydney. Two of these focus groups were conducted by staff from a local health district (LHD) and eight by four bilingual community research assistants (BCRAs). The BCRAs were members of the Arabic speaking communities and received training from the research team in how to conduct focus groups. The training session emphasised the need for a reflexive approach to the conduct of focus groups. People’s personal values were discussed and put to one side to ensure that a non-judgmental and open dialogue could be achieved in the focus groups. The eight focus groups conducted by BCRAs were in Arabic and/or English, and the two focus groups conducted by members of the research team were in English.

### Recruitment

The four BCRAs recruited participants and facilitated two focus groups each during a three month period (August to October 2016). Focus group participants were recruited from Arabic-speaking communities in an area of metropolitan Sydney. A convenience sampling approach to recruitment was undertaken. The BCRAs contacted existing community groups, including women’s groups, non-government organisations, local gyms and youth groups as well as networks of family and friends, via, phone calls, invitation flyers and promotion through the local mosque, Islamic centre and social media. Participants had to be a member of an Arabic-speaking community and over the age of 18 years. It was not intended that the focus groups be based on particular demographic characteristics. Both men and women were recruited. All participants who expressed interest in participating in the focus groups were included.

### Focus group questions

Focus group questions are shown in Table 1. These were informed by a rapid review of the literature and compilation of a list of potential health promotion interventions (community action; health information and social marketing; skills development and health education; screening, individual risk assessment; setting and supportive environment) [17]. The focus groups questions were used by the BCRAs as a guide and as prompts for opening the discussion with the participants. The focus group questions were written in English. The BCRAs were fluent in both Arabic and English and were able to verbally translate key concepts as needed during the focus groups.

**Table 1:** Focus group questions

1. What does smoking waterpipe mean to people in your community and their families? 2. In a “usual” week how often would people in the community you know smoke water pipe? 3. What does “social use” mean? 4. What do people know about, and how do they understand, the health aspects of smoking waterpipe? 5. What sort of intervention would be acceptable/not acceptable to reduce waterpipe smoking in the community? 6. Thinking about the following types of interventions, which ones would be acceptable and which would not? Why?	

Information on the demographic characteristics of participants were recorded including gender, age group, country of birth and languages spoken. BCRAs were provided with a template for documenting the data and received training from the research team on what type of data to record and how to record it. Notes and relevant quotes were documented during the focus group by a note taker assisting the BCRAs. BCRAs were fluent in both Arabic and English and either documented or translated the notes into English. Some focus groups were audio recorded with the agreement of the participants. However, participants in some groups were uncomfortable with audio recording so only written notes were taken. Refreshments were provided and participants did not receive any incentives for participating.

### Data analysis

Two researchers read and coded the focus group notes. QSR International’s NVivo 11 Software was used to assist with the data management, including the coding and analysis [18]. Notes were entered into NVivo 11 and examined by two researchers. The first researcher (RK) coded and analysed the notes in NVivo 11, and the second researcher (MS) conducted manual coding from the printed notes.

Coding was done by free-coding [19] with reference to the questions outlined in Table 1 and the study’s overall research questions. These free codes were then grouped into concepts, i.e. sub-themes, and then themes. A list of themes and subthemes was generated and then extracted with the relevant data into tables. The data was further examined for common themes across the groups, and for points of divergence. Emerging themes were cross checked with the themes manually coded by the second researcher (MS) for consistency.

Data from individual focus groups on interventions were classified according to the dimensions outlined above [17]. A list was compiled of potential interventions and examined for acceptability among participants of different ages and gender.

The validity of findings were discussed by the research team and then presented at a meeting with three of the four BCRAs to validate and contextualise the key findings. Notes from this meeting were included in the final analysis.

A more detailed description of the study, using the RATS qualitative research review guidelines, is provided in Additional file 1 [20].

## Results

Ten focus groups, including 88 participants ranging in age from 18 to 60+ years, were conducted. Some focus groups were formed from established community or family groups where participants had existing relationships. Other groups came together specifically for the purpose of the focus group and participants may or may not have had existing relationships.

### Demographic characteristics of focus groups

Participants’ characteristics, including gender, age group, country of birth and languages spoken, are presented in Table 2. All participants were aged 18 years and over and all but one were members of the Arabic-speaking communities of interest to the research project. One participant of Nepalese background was part of an Arabic speaking youth group and was therefore included in the focus group. There were a similar proportion of male and female participants, and participants from a range of age groups were included. Participants were not selected on the basis of their smoking status and information about the individual smoking status of the participants was not collected.

**Table 2:** Characteristics of focus group participants (*n* = 88)

Characteristic	Category	n (%)
Gender	Female	45 (51%)
	Male	42 (48%)
	Not recorded	1 (1%)
Age (years)	18–25	40 (46%)
	26–35	31 (35%)
	36–50	14 (16%)
	51–60	1 (1%)
	60+	1 (1%)
	Not recorded	1 (1%)
Country of birth	Australia	59 (67%)
	Lebanon	20 (23%)
	Egypt	2 (2%)
	Iraq	2 (2%)
	Bahrain	1 (1%)
	Libya	1 (1%)
	Nepal	1 (1%)
	Sudan	1 (1%)
	Syria	1 (1%)
Languages spoken by participants	Arabic and English	60 (68%)
	Arabic only	7 (8%)
	English only	20 (23%)
	Nepali and English	1 (1%)

The number of participants in each focus group ranged from six to 16. There were groups of mixed gender, male and female only, youth and young adult participants. The majority of participants were aged between 18 and 35 years (82%) and 68% of participants spoke both Arabic and English. An additional file provides more detailed information about the characteristics of the participants and the members of each focus group [see Additional file 2].

### Thematic analysis

Seven key themes relating to the perceptions of waterpipe smoking (practices, cultural identity, acceptability, social connectedness, knowledge and perceptions of harm, trend and fashion, availability and access) were identified from the data. A further four themes, based on the classification of potential health promotion interventions acceptable to this community (health information and social marketing, health education, policy and legislation, intervention target groups and messages), were examined.

### Perceptions of waterpipe smoking

#### Waterpipe smoking practice

Participants reported that waterpipe smoking was widely practiced in the community across age groups and genders and that use was increasing, particularly in younger age groups. Use occurred particularly within social contexts such as people’s homes or in restaurants or parks, often after dinner with coffee and dessert.

“*like last week we went and had dessert and argile…”* (Focus group (FG) 8).

*“Not something you smoke alone often”* (FG2).

Waterpipe was perceived as being a family orientated or *“generational practice”* (FG7) passed on through families, where it was described as being customary. One group suggested that use was more widespread amongst families for whom waterpipe smoking was perceived as being part of the family tradition (FG10).

Unlike cigarettes, waterpipe smoking sessions were reported to continue for several hours with the waterpipe shared amongst smokers. Although less common, some groups mentioned examples of individuals who smoked the waterpipe alone, outside of social contexts, sometimes multiple times a day. The waterpipe was also described as being a routine practice, but the frequency of use varied from weekly to daily.

*“Smoking Arghili is a great way to unwind after a long day at work. It relaxes me. It is part of my routine”* (FG1).

#### Cultural identity

The waterpipe was consistently described by participants as being an expression of Arabic tradition and culture and for many participants it was perceived as a way of connecting with Arabic culture.

*“It’s a way of connecting to my culture- I feel like I am a part of my culture when I smoke the waterpipe - love that feeling”* (FG6).

For some older people, growing tobacco in their country of birth had been part of their community and livelihood. One group described how the practice had developed and been inherited across generations, from being smoked primarily by older men to becoming popular amongst women and younger men.

#### Social and cultural acceptability

Waterpipe smoking was perceived as a social activity permitted within Arabic-speaking cultures and by some religious leaders. It was described as being more acceptable than other social activities, such as drinking alcohol, and this acceptability had contributed to its widespread use. The social acceptability of women participating in waterpipe smoking was reported to be increasing, and that children were regularly exposed to the practice because it is commonly smoked at home. However, it is usually smoked outside so the extent to which family members, including children, are exposed to waterpipe smoke is uncertain.

Some groups reported that parents allowed adolescents to participate in waterpipe smoking, either through its preparation, or by allowing them to smoke at home. Waterpipe smoking was also perceived by parents as a preferable, more acceptable, source of entertainment for young people than tobacco, alcohol and other drug use and going out to nightclubs.

*“Young people and women in our community believe that people, as well as parents, think cigarette smoking is bad for a woman and teenager, but they have a more positive view about the Arghili, and as a result they smoke Arghili in the community or in the presence of their family members.”* (FG1).

#### Social connectedness

Waterpipe smoking was strongly associated with socialising, and perceived as a way of engaging with people and making friends. It wasn’t just the act of smoking that was described as being important and enjoyable, it was about connecting with family and friends. Some participants reported feeling socially excluded if they didn’t participate in waterpipe smoking.

*“Whatever the setting, the waterpipe enhances the social atmosphere. It is an inclusive activity that involves passing the waterpipe around and continues until late at night.”* (FG1).

*“It’s a time where you get together with your friends, family, acquaintances…”* (FG2).

One group described smoking waterpipe as creating a social space or opportunity to meet and catch up with multiple people at once, particularly for people who are time poor or have large families.

*“That’s the only way I can see my many cousins I don’t have time to go visit each one separately so we meet after dinner at a restaurant where they want to smoke, and that is how I get to see them.”* (FG7).

Smoking waterpipe was also linked to relaxation, winding down and relieving stress. Some young, female participants talked about using it to address an emotional need such as depression, and suppressing appetite for weight loss.

*“If something dramatically in your life has changed, and you need to fill that time, that gap, if you were caring for someone who is no longer around, the relationship is no longer there…”* (FG8).

#### Knowledge and perception of harm

Most groups described a lack of concern, knowledge and/or awareness in the community about the potential harms and health effects associated with waterpipe smoking. They reported a number of misconceptions or myths including a strong perception that waterpipe was comparatively less harmful than other types of smoking or drug use such as cigarette smoking and alcohol.

The filtration of the tobacco through water, smooth texture of the smoke, and the marketing of organic fruit flavourings appear to support the belief that waterpipe smoking isn’t dangerous, or is a safer alternative than cigarette smoking.

*“Fruit flavour makes it less harmful. I don’t believe it’s as harmful as cigarettes.”* (FG1).

*“It just never seemed like a health risk. I mean, the tobacco was flavoured by organic apples, watermelon, or pears. And organic stuff is always healthy.”* (FG1).

Participants talked about the omission of waterpipe smoking from the legislation, policy and health promotion campaigns that address cigarette smoking, and how this reinforces the perception that it must be less harmful and not dangerous. As noted above, waterpipe was perceived as being a comparatively safe or more positive source of entertainment for teenagers and young people that keeps them at home and out of trouble.

#### Trend and fashion

Part of the appeal of waterpipe smoking, particularly for young people, was about being on-trend, fashionable and fitting in. These perceptions may be influenced by the media and advertising which portray positive messages and images about waterpipe smoking. Groups reported that young people promote their waterpipe smoking activity on social media. For women, the waterpipe was seen as being equivalent to a fashion accessory, almost like clothing (source: BCRA validation meeting), and innovations in the design of the waterpipe apparatus and tobacco flavours contributed to increased use.

*“I use an apple and pineapple as the head and I put tobacco in that. It’s awesome.”* (FG5).

#### Availability and access

Participants reported that waterpipe smoking has become widely available and easy to access, particularly in cafes and restaurants. Smoking waterpipe at home provides an affordable alternative to going out. Waterpipe equipment is portable, compact and easy to assemble and home delivery services have made setting up the apparatus more convenient. The equipment and tobacco can be purchased through the internet, shops, and imported from overseas. Despite being expensive, the waterpipe is perceived as cheaper in comparison to cigarette smoking.

*“It costs a lot of money at a restaurant; it’s cheaper to do at home”.* (FG10).

*“They deliver it to your house in Lebanon for six thousand Lebanese, it is like 4 or 5 dollars”*. (FG9).

#### Acceptable health promotion interventions

A range of views were expressed about the acceptability of different interventions. The majority of suggested interventions related to health information and social marketing, health education, and settings and supportive environments, particularly in relation to using legislation and policy to influence waterpipe smoking practices [17].

#### Health information and social marketing

The suggested interventions were primarily about increasing community awareness regarding the health effects of waterpipe smoking. These included use of the media, such as television, radio and community newsletters, as well as using signs in mosques, Islamic and community centres, health warnings on café/lounges tables, engaging Arabic organisations and religious leaders, and utilising community, fundraising and social events. A young men’s group preferred using media influence to increase understanding about waterpipe use, rather than introducing restrictions.

Social media and online messages were perceived by all groups as acceptable strategies, particularly for younger people. Facebook, Twitter, WhatsApp, Instagram and YouTube were the social media platforms suggested. Participants indicated that short, focused social media messages would be more effective than extensive written information. One group of young people mentioned that older people in the community were using Facebook and that social media strategies may be acceptable for this group. Brochures in doctor’s surgeries were also suggested by women as an acceptable strategy to reach older people.

*“If it’s going to target the youth and adolescents, social media is the way to go.”* (FG8).

#### Health education

Various health education strategies were suggested for increasing knowledge and understanding about the health effects and harms of waterpipe smoking. These included campaigns, school education programs, community presentations and government interventions. Many groups suggested presenting examples of the effect on health, including “real life” stories and case studies that people could connect with, as well as providing statistics and evidence for the impact of waterpipe smoking on health.

*“There needs to be education first. Starting with evidence about the harmful effects of WPS.”* (FG10).

Young men felt that smoking education was ineffective and use of media, film and the arts would be more effective. Another group suggested that interventions should be linked with existing interventions for cigarette smoking to strengthen the link between waterpipe smoking and tobacco.

#### Policy and legislation

Attitudes toward the acceptability of using policy and legislation to restrict waterpipe smoking practices varied considerably. Young men said that restrictions affecting social gatherings were unacceptable. Opinions about banning waterpipe smoking in public places, such as parks, cafes and restaurants, were divided. Some women supported bans in public areas, particularly around children and in parks. A group of young adults felt bans were excessive and that restrictions in restaurants were also unacceptable. One group suggested a ban on advertising tobacco flavouring. The reasons given to explain the opposition of some participants to bans and restrictions on waterpipe in public places included the perception that such restrictions are *“oppressive”* or *“going overboard”,* that smoking waterpipe is a personal choice, and an activity that doesn’t affect the public.

*“It’s just a social thing and it’s not affecting anyone in a negative way”.* (FG2).

Other participants suggested that restrictions wouldn’t reduce prevalence or prevent the growing trend of waterpipe smoking because people smoke at home or in parks and are often introduced to waterpipe in those settings.

The perceived effectiveness of warning labels and packaging varied. One young adults group thought labelling (using images) on waterpipe tobacco was ineffective because they aren’t always seen when purchased in cafes. Another similar group was surprised there were no warnings and thought plain packaging should also be introduced as for cigarettes. Other groups suggested labelling was an acceptable intervention.

Increasing taxes and the cost of waterpipe tobacco were discussed in four groups with most suggesting it is an acceptable intervention. Heavy fines and taxes were perceived as unacceptable by young men.

#### Intervention target groups and messages

Parents, youth, young people and university students, were suggested as appropriate target groups for health interventions to address waterpipe smoking. Engaging religious leaders to promote the harmful effects on health was also perceived as important.

Most groups indicated that interventions should focus on the health implications and/or physical effects of waterpipe smoking. For parents, information about health effects could challenge cultural norms and change behaviours that encourage waterpipe smoking among young people as part of family tradition, or as an acceptable alternative to other forms of entertainment or drug use. This may influence the behaviour of young people who appear to smoke for reasons associated with cultural identity and social connection rather than rebellious or risk taking behaviour.

For young people, interventions that address the perceptions of glamour, normalisation and cultural identity by linking the desire to look good with positive health behaviours and highlighting the negative effects that waterpipe smoking can have on appearance were suggested. One group discussed young people and women who go to the gym because they want to look good, and suggested the use of targeted messages about the health effects of waterpipe and how smoking can diminish the effort and hard work they put in at the gym to achieve that. A second group suggested targeting messages to teenagers about the negative visual effects of smoking on the body, for example on the face and on beauty, as well as the negative impact on athletic ability.

Some participants described a significant need for interventions to address waterpipe smoking use in the community and more information about associated harms. Our findings showed that some participants perceived other issues, such as alcohol use, as being more important to address. Other participants felt that because the waterpipe is part of tradition, health promotion interventions wouldn’t work and could be perceived as bullying or a criticism on cultural practices (FG2–4).

*“I think no intervention will work. It’s part of tradition and we love it.”* (FG6).

*“It’s a bully tactic to ban waterpipe smoking in public places”* (FG2).

## Discussion

This is the first Australian study we are aware of that has attempted to explore the perceptions and cultural meaning of waterpipe smoking in Arabic-speaking communities. Waterpipe smoking was found to be widely practiced across age groups and genders. Factors which appear to support this widespread use include the increased accessibility of waterpipe both at home and in restaurants and cafes, a perceived lack of associated harm and the fact that it has become increasingly trendy and fashionable, particularly among young people. These findings are consistent with a previous review which demonstrated similar findings where youth held favourable perceptions of waterpipe smoking as attractive and fashionable [21].

The findings of this study also suggest there are a number of fundamental, interrelated factors underpinning waterpipe use that should be considered in the development of health promotion interventions. In the Arabic-speaking communities included in this study, the family appeared to have a significant role in passing on the practice of waterpipe smoking, and enhancing its social and cultural acceptability. Previous studies have shown that waterpipe smoking among school and university students is associated with smoking in the house and with family and friends [8]. The review by Akl et al. [21] found that families had a role in both promoting and discouraging waterpipe use suggesting that families may have a significant influence on passing the practice to the next generation. Targeting parents and the family environment may be particularly important as it appears to be the setting where children and young people are first exposed to waterpipe smoking as an acceptable form of socialising, relaxation and entertainment.

Waterpipe smoking seemed to have a different meaning to people than smoking cigarettes. It was perceived as a less harmful, more acceptable and was connected with socialising and relaxation. Our findings support those of previous research which indicates that although people believe there are negative health effects from waterpipe smoking, it is perceived as being less harmful than cigarette smoking [3]. These perceptions were also linked with the idea of the water having filtering properties and the fruit flavourings as in previous studies [3]. This study highlighted the misconception of harm that exists among parents who perceived that waterpipe smoking was a safer alternative for teenagers and young people than other forms of entertainment and drug use. There is a need for interventions to focus on debunking the myths that contribute toward this reduced perception of harm in comparison to cigarettes, and de-glamourisation of the practice, particularly among young people.

The mixed opinions found between and within the groups about the need for health promotion interventions, policy and legislation to address waterpipe smoking may be related to the level of understanding in the community about harmful effects, as well as the degree of cultural and traditional significance attached to smoking waterpipe that for some people, made it exempt from health interventions. The readiness of the community should be taken into consideration in determining the types of interventions that will be successful and acceptable. The groups highlighted a need to raise community awareness about the harms of waterpipe smoking and focused their discussions around health information and education strategies. This suggests that these approaches, backed by community leaders, may be required before other more personalised education interventions are likely to be successful. The connection of waterpipe smoking with Arabic culture indicates a need for a culturally sensitive approach to the development and implementation of health promotion interventions.

The findings suggest a number of implications for the development of health promotion interventions to address waterpipe smoking in Arabic speaking communities. Interventions that raise general awareness in the community about the harms of waterpipe smoking, using approaches and interventions that link the health effects of waterpipe smoking with the dangers of cigarette smoking already well-recognised and promoted, could be an appropriate starting point to begin shifting community attitudes and understanding. Parents and young people appear to be the most important target groups for these initial health promotion messages that could aim to debunk the existing myths about waterpipe smoking being a safer alternative for socialisation. These groups have an opportunity to develop alternative forms of socialisation, cultural expression and connection within their families and communities.

As stated previously the connection of waterpipe smoking with Arabic culture indicates the need for a culturally sensitive approach to the development of health promotion interventions that are locally developed and directed. Cultural competence requires understanding from health care practitioners about the significant influence of social and cultural factors on the health beliefs and behaviours of patients, and the consideration of these factors in the development of interventions [22]. Co-design has been identified as a key feature of community engagement which involves service providers and service users working in equal partnership [23]. Engaging community leaders, champions and members of the community in the co-design of interventions is one approach that health promotion organisations are using to ensure that interventions are culturally relevant and acceptable to the community. This includes working in partnership with non-governmental and community organisations who work closely with Arabic speaking communities.

There are some limitations that should be taken into consideration when interpreting the findings of this study. Firstly the study findings reflect the perceptions held by members of Arabic-speaking communities in one area of metropolitan Sydney and may not be representative of other communities or applicable to their context. Whilst all age groups were represented in the study sample, the majority of the study participants (82%) were aged between 18 and 35 years. The perceptions held and issues raised by the focus groups may not be entirely representative of the whole community and there may be other areas that need to be considered.

## Conclusions

Waterpipe smoking in the Arabic-speaking communities included in this study is related to feelings of cultural identity and belonging. There are many misconceptions about harm and limited information is available to warn people of health effects. Health education is needed to debunk myths and raise awareness of potential harms. Our findings suggest that until and unless waterpipe smoking is perceived as a problem, community readiness for accepting interventions will be limited.

## Additional files

Additional file 1: Description of this study against qualitative review guidelines – RATS. This table provides a description of the study against guidelines for the review of qualitative research. These guidelines include the relevance of the study question, appropriateness of the qualitative method, transparency of procedures and the soundness of interpretive approach. (PDF 233 kb)

Additional file 2: **Table S1.** Characteristics of focus group participants. This table provides more detailed information about the characteristics of the focus groups included in the study. The focus group characteristics include the number of participants, participant gender, age groups represented, language(s) spoken by the participants and participant country of birth. (PDF 94 kb)

## Abbreviations

BCRA: Bilingual community research assistant
FG: Focus group
LHD: Local health district
