# Supplementary material for: Shaping interventions to address waterpipe smoking in Arabic-speaking communities in Sydney, Australia: a qualitative study
Source: BMC Public Health. 2018 Dec 17;18:1379. doi: 10.1186/s12889-018-6270-3 (PMC6296050; doi:10.1186/s12889-018-6270-3)
Supplement: Supplementary file 1 — Description of this study against qualitative review guidelines – RATS. This table provides a description of the study against guidelines for the review of qualitative research. These guidelines include the relevance of the study question, appropriateness of the qualitative method, transparency of procedures and the soundness of interpretive approach. (PDF 233 kb) [file 12889_2018_6270_MOESM1_ESM.pdf]

Additional file 1: Description of this study against qualitative review guidelines – RATS[1]

Ask of the manuscript	This paper
**R Relevance of study question**	
Is the research question interesting?<br><br>Is the research question relevant to clinical practice, public health or policy?	Research questions:<br><br>What are the perceptions held by Arabic-speaking communities about the extent of waterpipe use in their community; the cultural factors underpinning the use of waterpipe in the community; and community concern and knowledge of harm about waterpipe smoking?<br><br>What are the health promotion interventions that would be acceptable for addressing waterpipe smoking in an Arabic-speaking community?<br><br>A community consultation process undertaken by a local health district (LHD) prior to this research indicated that waterpipe smoking was a widespread and accepted practice in Arabic-speaking communities in an area of Sydney, Australia. It also identified that there was a concern within these communities about increasing use and a lack of knowledge of potential harms associated with waterpipe smoking.<br><br>A literature review conducted by the researchers prior to this qualitative study explored the evidence base on health promotion interventions to address waterpipe smoking. The evidence base was limited and there were no Australian studies identified by the review.<br><br>The research questions are relevant to both the local communities with whom the research was conducted, and are of interest to public health and health promotion practitioners/researchers.
**A Appropriateness of qualitative method**	
Is qualitative methodology the best approach for the study aims?	This study included ten focus groups with 88 participants in total.<br><br>Focus groups were an appropriate methodology to investigate the research questions, which were about the perceptions held about waterpipe smoking by the community rather than by individuals. Focus groups also allowed a larger number of community members to participate in the research and to add to each others' responses, with the aim of capturing a broader range of experiences and perspectives during the data collection.<br><br>Bilingual community research assistants (BCRAs) were chosen to conduct the focus groups in either Arabic or English. This allowed Arabic only speakers to participate in the research.
**T Transparency of procedures**	

[1] Clark J: How to peer review a qualitative manuscript. In Peer Review in Health Sciences. Edited by Godlee F, Jefferson T. London: BMJ Books; 2003:219–235.

Ask of the manuscript	This paper
Sampling	
Are the participants selected the most appropriate to provide access to the type of knowledge sought by the study?<br><br>Is the sampling strategy appropriate?	Participants were recruited from Arabic-speaking communities in an area of metropolitan Sydney. Participants had to be members of an Arabic-speaking community and over the age of 18 years to participate. There were no other selection criteria to participate in the study. Some groups were formed specifically for the purpose of the study. Other groups were made up of family members or groups already established in the community e.g. youth or women's groups.<br><br>This sampling strategy was appropriate to aims of the research as well as being appropriate to the cultural and linguistic needs of the participants.
Recruitment	
Was recruitment conducted using appropriate methods?	Study participants were recruited from the community by four trained BCRAs who were members of an Arabic-speaking community participating in the research. The BCRAs used a variety of methods to recruit participants including phone calls, invitation flyers and promotion through the local mosque, Islamic centre, social media, or directly from an established community group.
**Is the sampling strategy appropriate?**	
Could there be selection bias?	Focus group participants were all members of the communities of interest to the research project. There was a similar proportion of male and female participants, and participants from a range of age groups were included.
Data collection	
Was collection of data systematic and comprehensive?	Each focus group was asked the same set of questions:<br><br>1. What does smoking waterpipe mean to people in your community and their families?<br><br>2. In a "usual" week how often would people in the community you know smoke water pipe?<br><br>3. What does "social use" mean?<br><br>4. What do people know about, and how do they understand, the health aspects of smoking waterpipe?<br><br>5. What sort of intervention would be acceptable/not acceptable to reduce waterpipe smoking in the community?<br><br>6. Thinking about the following types of interventions, which ones would be acceptable and which would not? Why?
Are characteristics of the study groups and setting clear?	All participants were aged 18 years and over and part of an Arabic-speaking community. It was not intended that the focus groups be based on any other demographic characteristics. However, the recruitment strategy allowed

Ask of the manuscript	This paper
	for the BCRAs to recruit participants from groups already established within these communities and language groups. The number and characteristics of each group have been provided in Additional table 1.
Why and when was data collection stopped, and is this reasonable?	Each BCRA was asked to recruit participants and run two focus groups as part of their commitment to the research study. The number of focus groups and participants was appropriate for an exploratory qualitative study of this type.
Role of researchers	
Is the researcher(s) appropriate? How might they bias (good and bad) the conduct of the study and results?	The members of the research team came from the University of New South Wales, a LHD and the community. The team members provided the expertise in qualitative research methods, multicultural health, health promotion and local community knowledge required to conduct the study.<br><br>The BCRAs were recruited and employed by the LHD, and were responsible for recruiting the study participants and conducting the focus groups. The BCRAs were provided with comprehensive training by UNSW and LHD staff on participant recruitment, consent processes and conducting focus groups. Their knowledge of their local community meant they were appropriate for the role of BCRA.
Ethics	
Was informed consent sought and granted?	A participant information sheet was provided to participants. Written consent was obtained from participants. The participant information sheet and consent form were translated into Arabic for participants who did not speak English.
Were participants' anonymity and confidentiality ensured?	The anonymity of the participants was ensured in the participant information sheet. The names of the focus group participants were not documented with the focus groups notes. The quotes documented by the BCRAs in the focus group notes were not identifiable or attributed to individual participants.
Was approval from an appropriate ethics committee received?	Ethics approval was granted by a LHD Human Research Ethics Committee (HREC ref no: 16/143, approved 26/5/2016).
**S Soundness of interpretive approach**<br><br>***Analysis***	
Is the type of analysis appropriate for the study?<br><br>Are the interpretations clearly presented and adequately supported by the evidence?	Qualitative descriptive analysis was undertaken, with close reference to the focus group questions and the overall research questions. Coding was done using NVivo 11[2].

[2] QSR International Pty Ltd. NVivo qualitative data analysis software. Version 11. 2015.

Ask of the manuscript	This paper
Are quotes used and are these appropriate and effective?	Quotes have been used in the manuscript to provide examples from the data that illustrate the main research findings. Care has been taken to not include quotes that would enable study participants to be identified.
Was trustworthiness/reliability of the data and interpretations checked?	The research findings and interpretations drawn from the analysis were discussed and checked during meetings of the research team including the BCRAs who had conducted the focus groups.
Discussion and presentation	
Are findings sufficiently grounded in a theoretical or conceptual framework?	The Northern Territory Government Health Promotion Framework[3] was used to classify the health promotion interventions discussed by the focus group participants.
Is adequate account taken of previous knowledge and how the findings add?	Both the background and discussion sections draw on the findings of previous research. The discussion section of this paper describes how the findings presented are supported by previous research, as well as the implications for the future development of health promotion interventions in addressing waterpipe smoking in Arabic-speaking communities.
Are the limitations thoughtfully considered?	The limitations of this study are discussed in the manuscript.
Is the manuscript well-written and accessible?	The manuscript conforms with BMC Public Health requirements.
Are red flags present? These are common features of ill-conceived or poorly executed qualitative studies, are a cause for concern and must be viewed critically. They might be fatal flaws, or they may result from a lack of detail or clarity.	This study had a clear aim, clear research questions, approach to analysis, and obtained ethics approval prior to being conducted.

[3] Northern Territory Government Health Promotion Framework. Department of Health Darwin; 2013.
