# Supplementary material for: Shaping interventions to address waterpipe smoking in Arabic-speaking communities in Sydney, Australia: a qualitative study
Source: BMC Public Health. 2018 Dec 17;18:1379. doi: 10.1186/s12889-018-6270-3 (PMC6296050; doi:10.1186/s12889-018-6270-3)
Supplement: Supplementary file 2 — Table S1. Characteristics of focus group participants. This table provides more detailed information about the characteristics of the focus groups included in the study. The focus group characteristics include the number of participants, participant gender, age groups represented, language(s) spoken by the participants and participant country of birth. (PDF 94 kb) [file 12889_2018_6270_MOESM2_ESM.pdf]

Supplementary table 1. Characteristics of focus group participants (n=88)

Group	Number of participants	Gender	Age group (years)	Language(s) spoken in the focus group	Country of birth
**1. Mixed**	11	Female = 5<br>Male = 6	18-25 = 3<br>26-35 = 5<br>36-50 = 2<br>51-60 = 1	English with Arabic translation for some participants	Lebanon = 6<br>Australia = 3<br>Bahrain = 1<br>Iraq =1
**2. Young men**	9	Male = 9	18-25 = 6<br>26-35 = 3	Mainly English with some Arabic	Australia = 7<br>Iraq = 1<br>Lebanon = 1
**3. Young adults**	9	Female = 1<br>Male =8	18-25 = 8<br>Not reported = 1	Arabic and English	Australia = 9
**4. Mixed**	9	Female = 6<br>Male = 2<br>Not reported = 1	18-25 = 4<br>26-35 = 3<br>36-50 = 2	Arabic and English	Lebanon = 6<br>Australia = 2<br>Syria = 1
**5. Mixed**	8	Female = 5<br>Male= 3	26-35 = 4<br>36-50 = 3<br>60+ = 1	Arabic and English	Australia = 6<br>Lebanon = 2
**6. Young adults**	8	Female = 5<br>Male = 3	18-25 = 4<br>26-35 = 4	English	Australia = 8
**7. Women**	8	Female = 8	26-35 = 3<br>36-50 = 5	English	Lebanon = 4<br>Australia = 3<br>Egypt = 1
**8. Young women**	6	Female = 6	18-25 = 4<br>26-35 = 2	English	Australia = 6
**9. Young adults**	16	Female = 6<br>Male = 10	18-25 = 10<br>26-35= 6	English	Australia = 12<br>Egypt = 1<br>Libya = 1<br>Nepal = 1<br>Sudan = 1
**10. BCRA**	4	Female = 3<br>Male = 1	18-25 = 1<br>26-35 = 1<br>36-50 = 2	English	Australia = 3<br>Lebanon = 1
**TOTAL**	88	Female = 45<br>Male = 42<br>Not recorded = 1	18-25 = 40<br>26-35 = 31<br>36-50 = 14<br>51-60 = 1<br>60+ = 1<br>Not recorded = 1	English = 5<br>Arabic and English = 5	Australia = 59<br>Lebanon = 20<br>Egypt = 2<br>Iraq = 2<br>Bahrain = 1<br>Libya = 1<br>Nepal = 1<br>Sudan = 1<br>Syria = 1
